# A Comparison of Screening Tools for the Early Detection of Peripheral Neuropathy in Adults with and without Type 2 Diabetes

**DOI:** 10.1155/2017/1467213

**Published:** 2017-11-08

**Authors:** Jennifer J. Brown, Shana L. Pribesh, Kimberly G. Baskette, Aaron I. Vinik, Sheri R. Colberg

**Affiliations:** ^1^Elizabeth City State University, Elizabeth City, NC, USA; ^2^Old Dominion University, Norfolk, VA, USA; ^3^Eastern Virginia Medical School, Norfolk, VA, USA

## Abstract

**Objective:**

Examine the effectiveness of the 128 Hz tuning fork, two monofilaments, and Norfolk Quality of Life Diabetic Neuropathy (QOL-DN) questionnaire as tools for the early detection of diabetic peripheral neuropathy (DPN) in overweight, obese, and inactive (OOI) adults or those who have prediabetes (PD) or type 2 diabetes (T2D).

**Research Design and Methods:**

Thirty-four adults (mean age 58.4 years ± 12.1) were divided by glycemia (10 OOI normoglycemic, 13 PD, and 11 T2D). Sural nerves were tested bilaterally with the NC-stat DPNCheck to determine sural nerve amplitude potential (SNAP) and sural nerve conduction velocity (SNCV). All other testing results were compared to SNAP and SNCV.

**Results:**

Total 1 g monofilament scores significantly correlated with SNAP values and yielded the highest sensitivity and specificity combinations of tested measures. Total QOL-DN scores negatively correlated with SNAP values, as did QOL-DN symptoms. QOL-DN activities of daily living correlated with the right SNAP, and the QOL-DN small fiber subscore correlated with SNCV.

**Conclusions:**

The 1 g monofilament and total QOL-DN are effective, low-cost tools for the early detection of DPN in OOI, PD, and T2D adults. The 128 Hz tuning fork and 10 g monofilament may assist DPN screening as a tandem, but not primary, early DPN detection screening tools.

## 1. Introduction

Diabetes can result in long-term health complications, with one of the most common being microvascular damage that leads to diabetic neuropathy (DN), which is an insidious, variated pathology that affects multiple body systems and increases amputation risk [1, 2]. A typical form of DN is diabetic peripheral neuropathy (DPN), which is known to be a primary cause of balance issues [2–4], sensation loss in the feet [5], and a major contributor to nontraumatic lower limb amputations [2]. This pathology is a particularly significant concern for individuals with diabetes, as it is common and often leads to disability, yet it is difficult to diagnose due to frequent asymptomatic onset or unusual presentation [1, 2, 6–8]. DPN affects the nerve endings in the feet, hands, and other regions of the body after an individual has experienced frequent or extended hyperglycemia or other pathologies that lead to the loss of various forms of sensation [9–11].

Earlier detection of DPN in at-risk individuals and in those with prediabetes (PD) or type 2 diabetes (T2D) allows for potential better management through optimal intervention and lifestyle changes [12–14]. Limited research has sought to detect subclinical changes utilizing expensive and nonportable nerve conduction units, but the discovery of early changes in sensation using readily accessible, portable tools has not been a primary focus [12, 15, 16]. DPN often develops silently over time, making early detection and intervention difficult [14, 17–19]. Earlier intervention would likely allow for more positive outcomes, and low-cost tools to detect symptomology before the diagnosis of PD or T2D may be useful. Several tools, such as the 128 Hz tuning fork and the 1 g and 10 g monofilaments, have been successfully used for screening and disease assessment in adults with PD and T2D [20–26]. The Norfolk Quality of Life Diabetic Neuropathy (QOL-DN) questionnaire, NC-stat DPNCheck, and hemoglobin A1C testing (HbA_1C_) have been validated in T2D and limited PD populations, making them likely candidates for successful early screening efforts [1, 27–31].

While each measure has been shown to be reliable and valid in adults with PD and T2D, overweight, obese, and inactive (OOI) populations are also at high risk for the development of T2D and associated complications [19, 32]. Therefore, the aim of this study was to evaluate the effectiveness of identifying early signs of DPN using the 128 Hz tuning fork, 1 g and 10 g monofilaments, and QOL-DN questionnaire in adults who were OOI or had PD or T2D.

## 2. Materials and Methods

### 2.1. Study Participants

Volunteer subjects were recruited by flyers, email, word of mouth, and university-wide announcements. Subjects were screened by phone for exclusionary factors prior to reporting for testing. Potential subjects then reported to the Old Dominion University Wellness Institute for additional screening, informed consent, and testing measures. Assignment to groups was based on current HbA_1C_ testing values obtained onsite during study procedures. Individuals with a history of type 1 diabetes, tobacco use, hepatitis B, hepatitis C, HIV, pregnancy, damage to the lower extremities, nerve disease (other than neuropathy), peripheral arterial disease, lower limb amputations, foot ulcers, or a serious medical condition that would compromise subject safety or the integrity of the study were excluded. Study procedures were approved by the Old Dominion University Institutional Review Board prior to recruitment, and subjects gave their informed consent before participating.

### 2.2. Main Objective

This study was designed to examine the effectiveness of the 128 Hz tuning fork, 1 g and 10 g monofilaments, and QOL-DN questionnaire for the identification of early signs of DPN in OOI, PD, and T2D study populations. All testing results were compared to the SNAP and SNCV of the NC-stat DPNCheck, which served as the criterion measure for the study.

### 2.3. HbA_1C_ Testing

Participants followed hydration instructions for 24 hours (8–10 cups of fluid) and 2-3 hours (2-3 cups of fluid) prior to appointment times to avoid point of care device (POCD) high total hemoglobin errors. Finger-stick testing was performed with a DCA Vantage 2000 Analyzer (Siemens, Tarrytown, NY) and DCA Vantage HbA_1C_ test kits utilizing sterile techniques [33]. HbA_1C_ values and prior diagnoses were utilized to screen and categorize subjects as follows: OOI 4.0–5.6%; PD 5.7–6.4%; and T2D 6.5% and above [15, 34, 35].

### 2.4. NC-stat DPNCheck

Nerve conduction study procedures utilized the POCD NC-stat DPNCheck (DPNCheck, NeuroMetrix Inc., Waltham, MA) and followed previously outlined methods [30]. The POCD test method utilized a validated, bilateral examination of the lower extremities to obtain SNAPs and SNCVs on large myelinated nerve fibers [30, 31, 36]. The device allows for evaluation by non-technologist personnel, assisting in DPN detection at significantly earlier stages when compared to bedside tests [31, 37, 38]. The device itself consists of four basic components: (1) biosensors, which are flexible, single-use units that facilitate nerve conduction, integrating temperature monitoring, nerve signal transmission, and the correct placement of the device; (2) an easy view monitor, which is lithium battery driven, displaying data collected from the biosensors; (3) a docking station that transmits data; and (4) an on-call information system that analyzes docking station data through proprietary algorithms, providing individualized reports.

Unique biosensor technology paired with the NC-stat DPNCheck unit's two probes allowed for quick onsite evaluation in a matter of minutes. After skin preparation, the probes were coated in conductive gel and applied directly to the skin, posterior to the lateral malleolus. With the single press of a button, the unit distributed 100 mA of current, which was detected by the single patient use disposable biosensor. A built-in thermometer accounted for variances in temperature between 23°C and 30°C and notified the operator of skin temperatures too cold for testing, preventing further use until appropriate temperatures were present. Up to five individual nerve conduction study attempts per leg were utilized to collect three sets of SNCV and SNAP values, each providing individualized feedback based on the patient's age, height, and weight data. Device errors were not recorded; however, zero readings were recorded by hand and reattempts were made up to the 5-trial limit, as individual tolerance permitted. When individuals could not tolerate the acquisition of 3 data points per leg, last observation carried forward (LOCF) methods were employed to complete the trial set [39]. Individual reports were generated and reviewed with each participant after the study, with referrals to appropriate professionals as needed. The provided interpretation guide for each report sets “normal” limits for each participant based on internal algorithms. Study participants were assigned “abnormal” nerve conduction study results if their personal results were lower than their personal calculated “normal” limit across both legs for SNAP or SNCV, indicating consistent and equal reduction across both limbs, or if the participant had bilateral, consistent reduction across both SNAP and SNCV values. The validity and effectiveness of the NC-stat DPNCheck system has been confirmed in prior research [31, 38]. This test served as a criterion standard for the study, and all other testing was compared to this measure.

### 2.5. QOL-DN Questionnaires

The QOL-DN, a validated instrument and method for assessing neuropathy and differentiating between autonomic, large, and small fiber impairments [1, 27, 29] through multiple subscore components, was utilized with each participant. Individuals were given the questionnaire in a quiet area of the testing facility where they could work undisturbed, at their own pace, until completion [1, 27–29].

### 2.6. Tuning Fork Testing

A 128 Hz tuning fork was used to assess vibration perception [40, 41]. Familiarization, site and method of testing, and all procedures for the “on/off” method followed standardized protocols as outlined by the Rapid Screening for Diabetic Neuropathy using the 128 Hz tuning fork [40–45] (the appendix). The timed tuning fork method was employed bilaterally, using the same methods as Perkins et al. [43]. The procedural execution of both sets of tuning fork tests for peripheral neuropathy was performed with the subjects lying in the supine position, with eyes closed during testing [43, 44].

### 2.7. Monofilament Testing

Commercially produced 1 g and 10 g monofilaments (North Coast Medical, San Jose, CA) were used with a standard lab testing table to evaluate sensation perception. Monofilament storage and testing took place in a temperature-controlled environment, within the parameters established by previous research [46, 47] (the appendix). Scheduling was spaced out over a period of six weeks, with fewer than 10 subjects per day, followed by a 1-day rest period before subsequent use. Monofilaments were utilized to assess sensation according to standardized guidelines [20, 44, 48]. Familiarization and testing procedures followed the Canadian Diabetes Association for the Rapid Screening of Diabetic Neuropathy guidelines for 10 g monofilament testing at the dorsum of the great toe, proximal to the nail bed. These procedures were applied to testing for the 4.17/1 g and 5.07/10 g monofilaments (North Coast Medical, San Jose, CA). Standardized procedures were used for familiarization, subject response patterns, sites tested, number of stimuli, and score assignments, with all testing performed with the subject lying supine, eyes closed, on a laboratory testing table, retaining shoes and socks until testing to maintain body temperature [20, 41, 43, 44, 48].

### 2.8. Statistical Analyses

Data analyses were performed using SPSS version 22.0 for Windows (SPSS, Chicago, IL). Participants, group characteristics, SNAP, and SNCV are presented as raw data. Criterion and dependent variable data were logarithmically transformed to best achieve normality for statistical analysis. Partial correlations were analyzed using Spearman's coefficients for the tuning fork, 1 g and 10 g monofilaments, QOL-DN and NC-stat DPNCheck results, to determine the relationship between the variables. Interpretation of nerve conduction studies and diabetic neuropathy across the study for means was based on a cutoff definition of <6 *μ*V for sural nerve amplitude potential, bilaterally [31]. Interpretation of nerve conduction studies was evaluated case by case through the NC-stat DPNCheck software report package, which provided individualized cutoff data for each participant to determine “normal” or “abnormal” status for evaluation, allowing for the transforming of data into dichotomous variables when appropriate. Age, HbA_1C_, and waist measurement (in cm) were controlled for within analyses by recoding nonparametric variables within SPSS before running statistical tests. ROC curves were calculated from dichotomous variables in SPSS that were developed from the participant data based on “rule in,” “rule out,” and total QOL-DN scoring. Kruskal-Wallis H tests were used to determine if there were differences between the three groups with pairwise comparisons using Dunn's (1964) procedure. Alpha was set at *p* < 0.05 for all analyses.

## 3. Results

In total, 34 adults (10 males, 24 females) of varying ethnicities [Caucasian (64.7%); African-American (12%)] participated in the study and were assigned to one of the three groups based on their HbA_1C_ values: 10 (29.4%) normoglycemic OOI adults (6 females, 4 males; 59.6 years, ±13.0 years), 13 (38.2%) with PD (11 females, 2 males; 56.4 years, ±12.2 years), and 11 (32.4%) with T2D (7 females, 4 males; 59.6 years, ±12.1 years). Group characteristics included mean weight (OOI: 87.93 kg, SD ± 10.94 kg; PD: 98.03 kg, SD ± 23.26 kg; and T2D: 101.30 kg, SD ± 17.68 kg), mean BMI (OOI: 30.90 kg/m^2^, SD ± 3.17 kg/m^2^; PD: 34.20 kg/m^2^, SD ± 6.71 kg/m^2^; and T2D: 35.10 kg/m^2^, SD ± 5.03 kg/m^2^), and mean HbA_1C_ (OOI: 5.3%, SD ± .36%; PD: 5.9%, SD ± .22%; and T2D: 7.8%, SD ± 2.12%). The ten males (29.4%) in the study had a mean age of 61 years (SD ± 13.53 years), a mean height of 1.745 m (SD ± .08 m), a mean weight of 105.90 kg (SD ± 20.62 kg), a mean BMI of 34.85 kg/m^2^ (SD ± 4.97 kg/m^2^), and a mean HbA_1C_ of 6.0% (SD ± .96%). Twenty-four females (70.6%) who participated in the study had mean group characteristics of the following: mean age of 57.2 years (SD ± 11.58 years), mean height of 1.66 m (SD ± .06 m), mean weight of 89.4 kg (SD ± 15.10 kg), mean BMI of 32.99 kg/m^2^ (SD ± 5.67 kg/m^2^), and mean HbA_1C_ of 6.5% (SD ± 1.79%).

Fifteen participants reported no prior diagnosis or knowledge of hyperglycemia. Five had PD (based on HbA_1C_). Without specific recruitment for OOI, 33 of the 34 subjects in all groups were overweight (9) or obese (24). Six individuals reported prior neuropathy diagnosis, while 28 individuals reported having no prior neuropathy diagnosis or knowledge. Ten reported T2D-specific medication usage as part of their personal medical plan, and two T2D subjects reported taking a combination of T2D and neuropathy medications.

Group means for SNAP and SNCV did not significantly vary by HbA_1C_ level (data not shown). No significant differences were evident among OOI, PD, and T2D groups on SNAP and SNCV values (SNAP: R *H*(2) = 1.460, *p* = 0.482 and L *H*(2) = 2.369, *p* = 0.306; SNCV: R *H*(2) = 1.874, *p* = 0.392 and L *H*(2) = 1.880, *p* = 0.391). Data means and standard deviations are presented (Table 1(a)). Twenty-seven individuals obtained confirmed, individualized, abnormal NCS results, of which 25 were bilateral and symmetrical (Table 2). Twenty-four participants presented with a combination of abnormal distal signs bilaterally, of which 2 also reported altered activities of daily living (ADLS) and 4 reported autonomic symptoms. Only 2 of the 24 reported changes in both ADLS and autonomic subscore features. One individual presented with no signs or symptoms. Seven cases presented with normal NCS findings, but in the presence of reported symptoms and reduced bilateral distal sensation.

**Figure 1:**
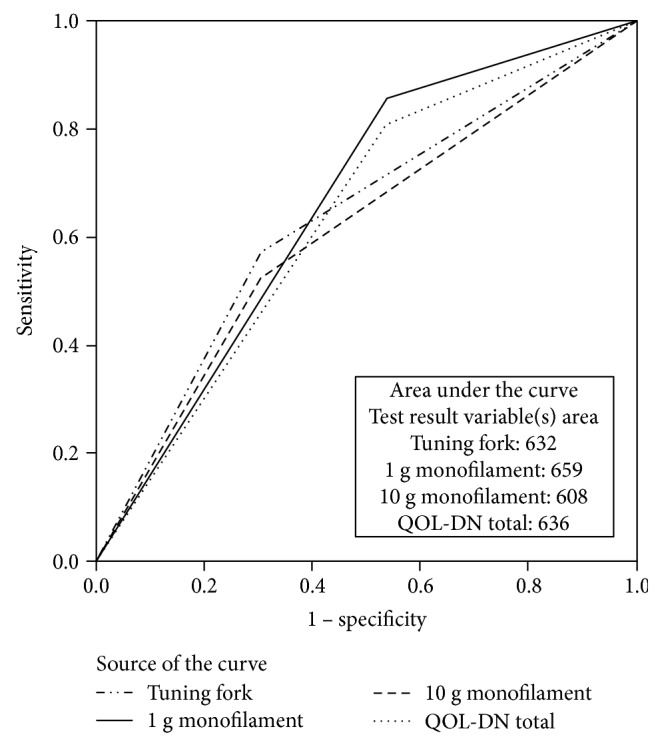
Receiver operator characteristics (ROC) curve.

The tuning fork on/off test did not correlate with criterion variables used in this study (see Table 1(b) and Table 3); however, the tuning fork achieved a sensitivity of 50.0%, specificity of 75.0%, and positive predictive value (PPV) of 69.2%. Timed tuning fork testing yielded no significant correlations or relationships within the study, bilaterally. The total 1 g monofilament scores moderately correlated with both SNAPs [R: *r_s_*(34) = 0.364, *p* = 0.024; L: *r_s_*(34) = 0.312, *p* = 0.047], and left 1 g monofilament scores demonstrated a moderate relationship to both SNAPs [R: *r_s_*(34) = 0.393, *p* = 0.016; L: *r_s_*(34) = 0.301, *p* = 0.053] and the left 1 g monofilament also correlated to the left SNCV (L: *r_s_*(34) = −0.313, *p* = 0.046) of the NC-stat DPNCheck. Sensitivity for the 1 g monofilament was 66.7% with a specificity of 72.0% and a PPV of 46.2% (see Table 1(b), Figure 1, Table 3).

The 10 g monofilament did not significantly correlate to criterion variables. Sensitivity for the 10 g monofilament was 47.4%, with specificity at 73.3% and a PPV of 69.2%. Total QOL-DN scores negatively correlated with both SNAPs [R: *r_s_*(34) = −0.317, *p* = 0.044; L: *r_s_*(34) = −0.311, *p* = 0.047], as did the QOL-DN symptom subscale (both SNAPs) [R: *r_s_*(34) = −0.332, *p* = 0.036; L: *r_s_*(34) = −0.375, *p* = 0.021], yielding a sensitivity of 60.0%, specificity of 70.8%, and PPV of 46.2% (Table 1(b), Table 3). The small fiber subscale of the QOL-DN correlated with the RSCV [R: *r_s_*(34) = −0.311, *p* = 0.047] and the QOL-DN ADLS subscale correlated with the RSNAP both SNAPs [R: *r_s_*(34) = −0.354, *p* = 0.028]. QOL-DN components spanned a wide range in sensitivity (36.4–60.0%) and specificity (60.9–100.0%), with PPV ranging from 30.8 to 100% (Table 1(b), Figure 1, Table 3).

## 4. Discussion

The integration of these testing methods provided foundational work necessary to develop a better understanding of the onset of dysfunctional physiological processes within OOI, PD, and T2D populations during the beginning of disease onset, shedding light on associations between symptoms and diseases. Moderate positive correlations were found between the 1 g monofilament and total and left leg scores with the recorded SNAP values. Additionally, the total QOL-DN, ADLS, and symptom scores negatively and moderately correlated to SNAPs, while small fiber scores negatively moderately correlated to SNCV. These correlations suggest that these tools may be useful for incorporation into low-cost screenings.

Detecting diabetes complications is an unfolding evolution that involves multiple dynamics to consider. DPN may present in a completely silent manner, without pain, burning, or symptoms of annoyance, and the utilization of the QOL-DN provides a unique, previously validated means of evaluating symptomology in at-risk populations [2]. Assessing DPN as early as possible is key, as individuals with early DPN may experience the disease in a varied manner with some individuals experiencing asymptomatic disease patterns, ultimately requiring hands-on screening to identify the silent progression of the disease. Pairing the QOL-DN with the 1 g and 10 g monofilaments and 128 Hz tuning fork provided us with an opportunity to explore previously validated tools in overweight, obese subjects who were inactive and at risk for the development of DPN.

This study utilized SNAP and SNCV values to evaluate nerve function in participants. Sural nerve conduction and amplitude values are validated quantitative physiological markers that assist in the assessment and confirmation of DPN status with or without the presence of signs or symptoms [30, 31]. Over 76% of participants in this study demonstrated abnormal NCS, 24 of whom reported symptoms and bilateral symmetrical signs upon examination that met the requirements for confirmed DSPN [49]; this is a significant percentage of participants in comparison to other research using the same measures [31]. Using low-cost screening tools, early DPN signs and symptoms were detected with a combination of methods that can be used on site, reliably, in a climate-controlled location. This is effectively different than other research attempts to identify early DPN, such as Mustafa et al., which required both blood work for C-reactive protein (CRP) and traditional NCS sural nerve evaluation [15]. Utilizing the NC-stat DPNCheck, the QOL-DN questionnaire, and QST measures, our investigation indicated subclinical neuropathy, a strong presence of early DPN signs, and pathology symptoms in the populations studied.

Other research has reported alternate findings, which may be at least partially explained by differences in the populations studied. Perkins et al. [31] only evaluated individuals with diagnosed diabetes (type 1 and type 2), whereas our study examined a wide range of subjects, including apparently healthy individuals recruited for our OOI population that may be prone to DPN, as well as adults with PD and T2D [31]. The bilateral, abnormal findings present in 71% of the individuals in this study have not been reported by others at a similarly high prevalence.

Assessment in this study differed from previous research by evaluating each individual participant according to age, height, and weight to determine appropriate cutoffs for normal and abnormal findings. This method individualized results to each participant with the built-in NC-stat software and included the potential impact of being overweight or obese. Having noted discrepancies between values of traditional NCS and the NC-stat DPNCheck, Lee et al. [30] performed a study that analyzed both measures, noting that the SNCV values tend to be lower with a traditional NCS when compared to the NC-stat DPNCheck. Given that traditional NCS is the ultimate “gold standard,” if the same type of error occurred in this study, it would likely boost the number of individuals who had abnormalities even higher.

To detect early DPN in normoglycemic OOI individuals, it had been hypothesized that the 128 Hz tuning fork and QOL-DN would provide the best mechanisms for detection; however, current results indicated only partial support for this. The tuning fork on/off test did not correlate well with the NC-stat DPNCheck SNAP criterion variables, although the QOL-DN did yield correlational results on several measures. This finding is different than some prior research, as the QOL-DN has not always been found to correlate with electrophysiological measures [50, 51].

The QOL-DN ranged in sensitivity (36.4–60.0%) and specificity (60.9–100.0%), differing from previous research that resulted in high specificity and sensitivity. The small fiber component weighed in with high specificity, yet overall, specificity averaged 60.9–75.0% with the other components. These results may have been affected by this study's population and its small number of subjects across three groups in attempting to uncover DPN at the earliest point possible. Previous research expressed concern relating to the QOL-DN: Hogg et al. [52] reported the QOL-DN as a means to aid in diagnosis and monitoring all types of diabetes neuropathy, but expressed a lack of specificity for PN, stating that it may be limited in its use to directly assess health impacts of a diabetes foot disease-related nature. Conversely, in this study, QOL-DN measures not only significantly correlated with DPN, but also provided vital standardized data relating to self-reported symptoms.

The 1 g monofilament proved to be a useful tool, with 31 individuals in this study experiencing abnormal findings. This measure indicated relatively high sensitivity (66.7%) and specificity (72.0%). However, validation of 1 g physical findings was achieved with moderate correlations back to the criterion SNAP variables, corroborating previous research that reported mixed sensitivity and specificity, such as Feng et al. and Taksande et al. [22, 53]. The 10 g monofilament testing lacked significant correlational relationships, yet the usefulness of this tool has been well established in T2D research and in limited PD populations by others. The findings in this study did not add support for its use in normoglycemic obese populations, but insensate feet relate to neuropathy in later stages and this research focused on early detection. Current findings did, however, somewhat parallel to Perkins et al. [43], who achieved lower sensitivity (53%) with the 10 g monofilament. In contrast, Ylitalo et al. [54] found that the 10 g monofilament was a useful tool for uncovering neuropathy in obese individuals, yet that study only examined women and allowed smokers and T1D subjects within their study cohort, possibly accounting for some differences in results.

The QOL-DN (S: 60.0%; SP: 70.8%) was hypothesized to be the most sensitive measure to detect undisclosed DPN in the study population; instead, the 1 g monofilament (S: 66.7%; SP: 72.0%) performed slightly better within this limited cohort. The 128 Hz tuning fork on/off test (S: 50.0%; SP: 75.0%) fell not too far behind. The QOL-DN proved to be lower in sensitivity in this study and ranged in specificity, yet many components (total QOL, symptoms, ADLS, and small fiber) correlated to the criterion measure used. Sample size was quite small, and with the significant previous validation history of the QOL-DN, it is likely that this screening instrument in its entirety may be useful in determining both small and large fiber deficits in a larger study in overweight, obese, and inactive individuals [1, 50, 51, 55]. The criterion measure, the NC-stat DPNCheck, targets screening for large fiber deficits and may not correlate as well with a well-rounded screening measure that targets multiple areas of neuropathy like the QOL-DN. Future research should likely continue to examine the QOL-DN for early DPN detection, as several subscales indicate significant correlations.

There is a strong indication of early-onset and subclinical neuropathy in the populations in this study, suggesting that careful screening of individuals at earlier stages may be quite beneficial in the early detection of DPN, even prior to hyperglycemia onset in OOI and PD. Smith and Singleton [56] found elevated HbA_1C_ status in such populations to be a concern for the development of large fiber-related neuropathy complications, in accordance with this cohort. Diabetes-related complications, such as decreased motor and sensory nerve conduction velocities, may arise out of acute bouts of hyperglycemia experienced though postprandial excursions, which may be best reflected by HbA_1C_ values [9].

This pilot study has some limitations. Besides being small, the cohort was limited to one site of investigation and only two ethnic groups; therefore, its findings must be placed within the context of the larger body of literature available. Lack of random assignment and use of volunteers for subjects created potential selection bias, with the use of clinical populations and low available funding heavily influencing these methods. HbA_1C_ testing was performed with a validated machine, yet oral glucose tolerance testing is preferred by some researchers, particularly for individuals with cardiac autonomic neuropathy (CAN) [57]. This study did not test for CAN and, therefore, cannot account for unknown discrepancies that could affect results. Temperature, humidity, and high-volume testing in short periods of time have been found to affect the validity of monofilament testing [46, 58]. Temperature was accounted for by limiting monofilament storage and use to normal climate-controlled room temperatures and the monitoring of these values. While humidity was monitored, it was not controlled beyond what the laboratory air conditioning and heating systems accounted for. Monofilament usage followed previously stated guidelines and recommendations, with combined testing amounting to less than 100 compressions per day per instrument [58]. The NC-stat DPNCheck device was used solely to test the sural nerve, as in the case of Perkins et al. [31]; therefore, deficits in nerve function relating to other nerves of the lower leg were not confirmed through this device and two nerves were not evaluated, as some literature advises. The QOL-DN has been previously validated for individuals with diabetes and neuropathy, yet its specific validation to effectively target OOI individuals has not been performed and, therefore, this should be taken into account when interpreting the findings.

Although we applied rigorous testing preparation and methods, it is possible that there was an error we remain unaware of that affects the validity of these findings [59]. It is also possible that the NC-stat DPNCheck current software components and algorithms are too sensitive for the subject population. For clarification, we compared our SNAPs to Perkins et al. and found that, overall, SNAP values in this study contained values ranging from 2 to 25 *μ*V, with means ranging from 6.6 to 10.5 *μ*V, compared to Perkins et al., who contained means of 5.6 *μ*V. Many of their participants (16) had undetectable levels, whereas this study obtained three readings on all but 4 individuals to whom LOCF was applied. Acquisition of three readings on each leg across a diverse collection of individuals, all of whom were likely to develop DPN, likely increases the validity of these findings. In further support, the individuals with abnormal findings self-reported symptoms via QOL-DN symptoms subscale and had documented distal sensation loss via 128 Hz tuning fork and 1 g or 10 g monofilaments.

Unique features of testing done in this study include accounting for age, height, and weight individually, securing a minimum of 3 readings per leg, therefore allowing a more individualized and accurate assessment of the large myelinated nerve fibers. Small fiber-associated deficits were not directly assessed in this study. The testing done does offer a nonclinical analysis based on the criteria required by Tesfaye et al. aiming to achieve minimal definition requirements for confirmed and subclinical DSPN classification, with the intent of developing early screening measures for DPN-prone populations [49].

## 5. Conclusions

In summary, early DPN signs and symptomology can be detected in OOI, PD, and T2D populations using low-cost, established tools. The 1 g monofilament proved to be more useful for the early detection of DPN than the 10 g monofilament within this population by correlating to the study standard and providing the highest sensitivity and specificity combination. The total QOL measure also proved to be useful, correlating to the standard, yielding the second highest combination of sensitivity and specificity to SNAP and SNCV values. Several QOL-DN subscales (ADLS, Small Fiber, Symptoms) provided valuable, standardized information that can be incorporated into low-cost community screening models for early DPN detection in populations with or without PD and T2D, while providing varied sensitivity and specificity across multiple categories. The 128 Hz tuning fork did not prove to be quite as accurate in this population as the QOL-DN and should be used as a tandem measure when screening. Future research should consider a larger study with the same populations, aiming to continue to refine and develop screening methods targeted towards disclosing both symptomatic and asymptomatic DPN.

## Figures and Tables

**Table:** (a) Sural nerve amplitude potentials and conduction velocities

	*N*	Min	Max	Mean	Std. error	Std. dev.
SNAP-R (*μ*V)						
OOI	10	2	14.3	6.631	1.444	4.567
PD	13	2	24.7	7.691	1.674	6.037
T2D	11	2	25	9.875	2.133	7.076
SNAP-L (*μ*V)						
OOI	10	2.3	21.7	7.129	1.834	5.798
PD	13	3	21.7	7.277	1.186	4.277
T2D	11	3	21.7	10.572	2.064	6.847
SNCV-R (*μ*V)						
OOI	10	35.3	55.7	46.2	1.902	6.016
PD	13	30	57	48.2	1.871	6.747
T2D	11	35.3	57	45.5	1.816	6.022
SNCV-L (*μ*V)						
OOI	10	41.3	55	47.265	1.519	4.803
PD	13	43	55	49.637	1.072	3.865
T2D	11	37.3	57	46.876	1.946	6.455

**Table:** (b) Sensitivity and specificity of screening tests and subcomponents

	Prevalence	Sensitivity	Specificity	PPV^∗^	NPV^∗^
128 Hz tuning fork	52.90%	50.00%	75.00%	69.20%	57.10%
1 g monofilament	26.50%	66.70%	72.00%	46.20%	85.70%
10 g monofilament	55.90%	47.40%	73.30%	69.20%	52.40%
QOL-DN total	29.40%	60.00%	70.80%	46.20%	81.00%
QOL-DN symptoms	32.40%	36.40%	60.90%	30.80%	66.70%
QOL-DN large fiber	35.30%	58.30%	72.70%	53.80%	76.20%
QOL-DN small fiber	97.10%	39.40%	100.00%	100.00%	4.80%
QOL-DN ADLS	76.50%	42.30%	75.00%	84.60%	28.60%
QOL-DN autonomic	61.80%	42.90%	69.20%	69.20%	42.90%

^∗^Normoglycemic, PD, and T2D. PPV = positive predictive value; NPV = negative predictive value; based off R/L SNAP values. Prevalence indicates presence of findings for indications of neuropathy.

**Table:** (a) Sural NCS, signs, and symptoms

	Variable	Total	Group		
			OOI	PD	T2D
Sural NCS *N* = 34	Normal	7	1	3	3
	Abnormal^∗^	27	10	9	8

Signs *N* = 34	Tuning fork				
	Normal	14	3	6	5
	Abnormal^∗^	20	7	7	6
	Monofilaments				
	1 g				
	Normal	3	1	0	2
	Abnormal^∗^	31	9	13	9
	10 g				
	Normal	3	1	0	2
	Abnormal^∗^	31	9	13	9

Symptoms *N* = 34	None reported	11	6	1	4
	Reported^∗∗^	23	4	12	7

Autonomic *N* = 34	None reported	21	7	8	6
	Reported^∗∗^	13	3	5	5

ADLS *N* = 34	None reported	26	8	10	8
	Reported^∗∗^	8	2	3	3

NCS, sign & symptom combinations	AbNCS, signs & symptoms	17	3	9	5
	AbNCS, signs, or symptoms	9	5	1	3
	AbNCS, no signs, or symptoms	1	1	0	0
	NNCS, signs & symptoms	7	1	3	3

**Table:** (b) SNAP values

		Min	Max	Mean (SD)	Std. error
SNAP-R (*μ*V)					
NNCS	*N* = 13	7.300	25.000	14.022 ± 5.64	1.564
AbNCS^∗^	*N* = 21	3.618	5.202	4.41 ± 1.740	0.379
SNAP-L (*μ*V)					
NNCS	*N* = 13	11.050	17.138	14.096 ± 5.033	1.396
AbNCS^∗^	*N* = 21	3.898	5.524	4.712 ± 1.786	0.389

^∗^Bilateral testing; abnormal findings on at least one limb. ^∗∗^Self-reported on QOL-DN. AbNCS: abnormal nerve conduction study; NNCS: normal nerve conduction study. Part (a): AbNCS defined by NC-stat DPNCheck report values for SNAPs and SNCVs. Part (b): AbNCS defined by 6.0 *μ*V or less SNAPs.

**Table 3:** NC-stat DPNCheck Spearman's partial correlations (log transformed).

	SNAP-R	SNAP-L	SNCV-R	SNCV-L
	*N* = 34	*N* = 34	*N* = 34	*N* = 34
*Tuning fork*				
On/off	0.221	0.137	0.235	−0.089
*p* value	0.121	0.235	0.106	0.319
Timed R	−0.066	−0.019	−0.019	−0.099
*p* value	0.365	0.461	0.459	0.302
Timed L	−0.063	−0.052	−0.018	−0.081
*p* value	0.371	0.392	0.463	0.355
*Monofilaments*				
Total 1 g	0.364^∗^	0.312^∗^	−0.06	−0.141
*p* value	0.024	0.047	0.377	0.229
1 g R	0.229	0.206	0.024	0.077
*p* value	0.112	0.138	0.451	0.342
1 g L	0.393^∗^	0.301^∗^	−0.191	−0.313^∗^
*p* value	0.016	0.053	0.155	0.046
Total 10 g	0.098	0.088	0.032	0.03
*p* value	0.304	0.321	0.432	0.438
10 g R	0.096	0.16	0.005	−0.066
*p* value	0.306	0.2	0.489	0.364
10 g L	0.137	0.07	0.031	0.054
*p* value	0.235	0.356	0.436	0.388
*QOL-DN*				
Total	−0.317^∗^	−0.311^∗^	0.162	−0.117
*p* value	0.044	0.047	0.197	0.269
Symptoms	−0.332^∗^	−0.375^∗^	0.213	−0.003
*p* value	0.036	0.021	0.129	0.493
Large fiber	−0.297	−0.284	0.107	−0.163
*p* value	0.056	0.064	0.286	0.195
Small fiber	−0.241	−0.187	−0.311^∗^	−0.366^∗^
*p* value	0.099	0.161	0.047	0.023
ADLS	−0.354^∗^	−0.263	0.104	−0.065
*p* value	0.028	0.08	0.293	0.366
Autonomic	−0.236	−0.245	0.149	−0.044
*p* value	0.105	0.096	0.216	0.408

Accounts for HbA_1C_, age, and waist (cm). ^∗^Significance at *p* < 0.05.

## Appendix

### A. Procedures for Screening for Diabetic Neuropathy

#### A.1. Monofilaments, 1g and 10g

The participant was first shown the monofilament, by allowing them to see it and then gently touching their forehead with the instrument. They were instructed to close their eyes and say “yes” each time that the stimulus was felt. The monofilament was applied to the dorsum of the great toe, just proximal to the nail bed for approximately one second, 4 times per foot, in an irregular pattern in such a way that the stimulus could not be anticipated. Scoring: 0 was assigned for no perception, 0.5 substantially less perception and 1 for normal perception. Totals: 0–3, the presence of neuropathy is likely; 3.5–5 indicates that the onset of neuropathy within four years is high; 5.5 and above indicates low risk.

#### A.2. Tuning Fork – 128 Hz

The tuning fork was struck against the palm of the testers hand so that it would vibrate for approximately 40 seconds and then applied to the base of the forehead so that participants could understand the concept of the vibration sensation. After asking the patient to close their eyes, the tuning fork was placed on the bony prominence at the dorsum of the first toe, which is just proximal to the nail bed. The participant was asked to report when the vibration stopped, and the tester dampened the tuning fork with the other hand. Scoring: 1 point was assigned for each correct perception of (vibration “on” or “off”). The procedure was performed twice on each foot in such a way that the participants could not anticipate the testers actions. This is a “rule out” test for the presence of neuropathy and does not indicate risks related to future onset.

This is adapted from the Canadian Diabetes Association, Rapid Screening for Diabetic Neuropathy [44].

## Abbreviations

ADLS: Activities of daily living scores
CAN: Cardiac autonomic neuropathy
DN: Diabetic neuropathy
DPN: Diabetic peripheral neuropathy
HbA_1C_: Hemoglobin A1C
LOCF: Last observation carried forward
NPV: Negative predictive value
QOL-DN: Norfolk Quality of Life Diabetic Neuropathy
OOI: Overweight, obese, inactive
POCD: Point of care device
PD: Prediabetes
PPV: Positive predictive value
SNAP: Sural nerve amplitude potential
SNCV: Sural nerve conduction velocity
T1D: Type 1 diabetes
T2D: Type 2 diabetes
QOL: Quality of life.
